# Cost-effective genome-wide estimation of allele frequencies from pooled DNA in Atlantic salmon (*Salmo salar* L.)

**DOI:** 10.1186/1471-2164-14-12

**Published:** 2013-01-16

**Authors:** Mikhail Ozerov, Anti Vasemägi, Vidar Wennevik, Eero Niemelä, Sergey Prusov, Matthew Kent, Juha-Pekka Vähä

**Affiliations:** 1 Kevo Subarctic Research Institute, University of Turku, Turku, 20014, Finland; 2 Department of Biology, Division of Genetics and Physiology, University of Turku, Turku, 20014, Finland; 3 Institute of Marine Research, PO Box 1870, Nordnes, N-5817, Bergen, Norway; 4 Finnish Game and Fisheries Research Institute, Rakentajantie 3,PL 413, 90014, Oulun yliopisto, Finland; 5 Freshwater Resources Laboratory, Knipovitch Polar Research Institute of Marine Fisheries and Oceanography, 6. Knipovitch Street, 183767, Murmansk, Russia; 6 Centre for Integrative Genetics (CIGENE), Department of Animal and Aquacultural Sciences (IHA), Norwegian University of Life Sciences, PO Box 5003, Ås, Norway; 7 Department of Aquaculture, Institute of Veterinary Medicine and Animal Science, Estonian University of Life Sciences, 51014, Tartu, Estonia

**Keywords:** DNA pooling, Atlantic salmon, SNP, Allele frequency estimation, Allelotyping, Population genomics

## Abstract

**Background:**

New sequencing technologies have tremendously increased the number of known molecular markers (single nucleotide polymorphisms; SNPs) in a variety of species. Concurrently, improvements to genotyping technology have now made it possible to efficiently genotype large numbers of genome-wide distributed SNPs enabling genome wide association studies (GWAS). However, genotyping significant numbers of individuals with large number of SNPs remains prohibitively expensive for many research groups. A possible solution to this problem is to determine allele frequencies from pooled DNA samples, such ‘allelotyping’ has been presented as a cost-effective alternative to individual genotyping and has become popular in human GWAS. In this article we have tested the effectiveness of DNA pooling to obtain accurate allele frequency estimates for Atlantic salmon (*Salmo salar* L.) populations using an Illumina SNP-chip.

**Results:**

In total, 56 Atlantic salmon DNA pools from 14 populations were analyzed on an Atlantic salmon SNP-chip containing probes for 5568 SNP markers, 3928 of which were bi-allelic. We developed an efficient quality control filter which enables exclusion of loci showing high error rate and minor allele frequency (MAF) close to zero. After applying multiple quality control filters we obtained allele frequency estimates for 3631 bi-allelic loci. We observed high concordance (*r* > 0.99) between allele frequency estimates derived from individual genotyping and DNA pools. Our results also indicate that even relatively small DNA pools (35 individuals) can provide accurate allele frequency estimates for a given sample.

**Conclusions:**

Despite of higher level of variation associated with array replicates compared to pool construction, we suggest that both sources of variation should be taken into account. This study demonstrates that DNA pooling allows fast and high-throughput determination of allele frequencies in Atlantic salmon enabling cost-efficient identification of informative markers for discrimination of populations at various geographical scales, as well as identification of loci controlling ecologically and economically important traits.

## Background

Technological advances in polymorphism detection and genotyping have made the single nucleotide polymorphisms (SNPs) the marker of choice for many high density genotyping studies [1,2]. High-throughput microarrays containing assays for thousands of SNPs are becoming available for a number of non-model organisms [1-3], and being used more frequently in ecological and evolutionary studies, including population genetics studies e.g. [4-7], QTL identification e.g. [8], parentage determination e.g. [9-11], and mixed stock analysis e.g. [12-15].

Despite the recent technical advances, genotyping large numbers of individuals with thousands of SNPs remains prohibitively expensive for many research groups. Furthermore, many population genetic studies are based on population allele frequency rather than individual genotype data. Therefore, determination of allele frequencies from pooled DNA samples, i.e. ‘allelotyping’, has been suggested more than 30 years ago as a cost-effective alternative to individual genotyping (reviewed by Sham et al. [16]). Several studies have successfully used this approach in genome-wide association studies that compare the allele frequencies between cases and controls e.g. [17-23]. These studies have demonstrated satisfactory accuracy and repeatability, and the DNA pooling approach can reduce costs by as much as 100-fold depending on the number of samples [16,21,23].

While the allelotyping of DNA pools can substantially reduce the costs compared to individual sample by sample genotyping, this approach is not without disadvantages. First, various sources of error occur during the allele frequency estimation from DNA pools. According to Earp et al. [23], variation introduced to allele frequency estimates can be divided into four categories: (i) within array; (ii) between arrays; (iii) between independently constructed identical pools, and (iv) between pools constructed from different individuals of the same population (biological replicates). Therefore, in order to obtain reliable allele frequency estimates using DNA pooling it is important to evaluate the magnitude and relative importance of different sources of error [23,24]. In addition, DNA pooling generally does not provide information about haplotype frequency and despite recent computational improvements [25,26] resolving the phase ambiguity remains a challenge for large number of loci [27]. However, despite the popularity of DNA pooling in genetic association studies, only few studies to date have utilized allelotyping approach to characterize inter-population variation e.g. [28].

Here, we tested the usefulness of DNA pooling for a first time using an Atlantic salmon (*Salmo salar* L.) Illumina SNP-chip to obtain accurate allele frequency estimates for multiple Atlantic salmon populations and evaluated the importance of different sources of errors arising from allelotyping. First, we assessed the effect of DNA pool construction and between-array variations on allele frequency estimates. Subsequently, the effect of cluster separation scores (parameter that summarizes the separation of three genotype classes in the theta dimension), two alternative sources of theta (a value between 0 and 1 which defines the genotype; 0 = AA, 1 = BB, 0.5 = AB) and DNA pool size on allele frequency estimation were evaluated. Finally, two alternative quality control (QC) filters were tested to select optimal sets of SNP loci for subsequent population genetic analysis.

## Results and discussion

In total, 56 Atlantic salmon DNA pools from 14 populations were analyzed using an Atlantic salmon SNP-chip [29,30] carrying probes for 5568 SNP markers 3928 of which were bi-allelic. After excluding 1640 non bi-allelic markers and 31 bi-allelic loci due to low call rate (< 95%) (see Additional file 1, Figure S1a) the repeatability of allelotyping from DNA pools was tested for 3897 loci.

### Array- vs. pool-construction variation

**Figure 1:**
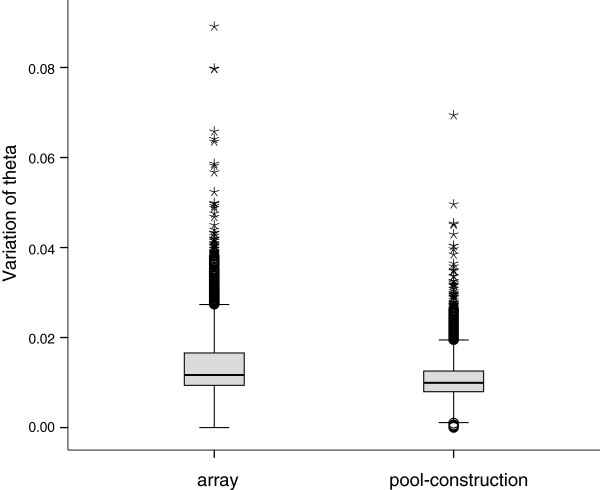
**Box-plot showing estimated array- and pool-construction variation of theta (Mann–Whitney *U*-test, *P *< 0.0001).** Horizontal line, grey square, whiskers, open circles, and stars indicate median, 25th and 75th quartiles, non-outlier range, outliers and extreme outliers, respectively.

The experimental design described in Table 1 provided 56 estimates of array-variation and 52 of pool-construction variation in the theta value. The mean array-variation per SNP varied from theta 0.000 to 0.089, whereas the mean pool-construction variation of theta ranged from 0.000 to 0.069. The estimated variation of theta between different arrays (i.e. array-variation using identical DNA pools) was 20% higher compared to variation arising from DNA pool construction (median_array_ = 0.012 vs. median_pool-construction_ = 0.010, non-parametric Mann–Whitney *U*-test, *P* < 0.0001) (Figure 1). These results suggest that it is more important to consider variation arising from different arrays than variation associated with pool construction [22-24,31]. This is in line with the earlier studies suggesting that running the same DNA pool in multiple arrays should be preferred over construction and analysis of multiple DNA pools within the same array [18,22,32]. However, considering the relatively similar levels of variation associated with the array and pool replicates, future studies should incorporate both sources of variation in the experimental design for reliable estimation of allele frequencies from DNA pools.

**Table 1:** Information about populations, their geographic locations, number of individuals and number of pool replicates studied

**Population**	**Number of individuals included in the pools and number of array and pool construction replicates (in brackets)**			**Number of samples for individual genotyping**
	**Pool-size 1**	**Pool-size 2**	**Pool-size 3**	
*Norwegian Sea coast*				
Alta		50 (2, 2)	70 (2, 2)	6
Laukhelle	35 (2, 0)	43 (2, 2)		5
Repparfjordelv		50 (2, 2)	69 (2, 2)	
*Barents Sea coast*				
Lakselva		50 (2, 2)	67 (3, 2)	
Vestre Jakobselv		50 (2, 2)	70 (2, 2)	
Tana Bru (Teno)		50 (2, 0)	60 (2, 0)	
Karasjoki (Teno)		50 (2, 2)	70 (2, 2)	
Inarijoki (Teno)		50 (2, 0)	67 (2, 2)	
Iesjoki (Teno)				6
Neiden		50 (2, 2)	63 (2, 2)	
Ura	35 (2, 0)	46 (2, 2)		
Titovka		50 (2, 2)	70 (2, 2)	
Kola	35 (3, 3)	50 (3, 3)	70 (3, 3)	67
Pechora Unya				6
*White Sea coast*				
Ponoi		50 (2, 2)	70 (2, 2)	
Varzuga		50 (2, 2)	70 (2, 2)	6
Onega				6
*Baltic Sea coast*				
Narva				4
***Total pooled***			905	
***Total individual***				106

### Estimation of allele frequencies from DNA pools

**Figure 2:**
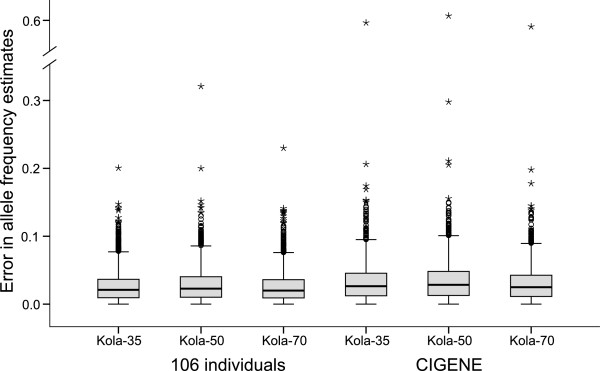
**Box-plot showing error in allele frequency estimates calculated using theta cluster mean values provided by CIGENE or obtained from 106 individuals (Mann–Whitney *U*-test, all tests, *P *< 0.0001).** Horizontal line, grey square, whiskers, open circles, and stars indicate median, 25th and 75th quartiles, non-outlier range, outliers and extreme outliers, respectively.

The allele frequencies for 3631 SNPs that passed the quality control (see below) were estimated from DNA pools using reference values of theta provided by CIGENE and reference values of theta derived from the genotyping of 106 individuals used in pool construction. Comparison of the two sets of theta values revealed a small, but significant, difference in allele frequency estimates. Using individual genotypes from this study to derive reference values of theta provided slightly higher accuracy in allele frequency estimates compared to the larger (n = 300) but unrelated dataset provided by CIGENE (median error_106_ = 0.020 – 0.023 vs. median error_CIGENE_ = 0.025 – 0.028; Mann–Whitney *U*-test, all tests, *P* < 0.0001; Figure 2). Errors associated with allele frequency estimations using reference values of theta from two different sources were significantly correlated (Pearson’s *r* = 0.640 – 0.684, *P* < 0.0001) suggesting that small number of SNPs suffer from larger error irrespective of the source of reference values of theta while the majority of loci have relatively low error rates. Taken together, these results suggest that even relatively small number of individuals (~ 100) is sufficient to generate reliable reference values of theta. However, because all three genotype classes are needed for accurate estimation of allele frequencies, using relatively small number of individuals resulted in loss of SNPs as not all genotypes were observed in the reference datasets (3631 vs. 3138 SNPs based on CIGENE and our data, respectively).

**Figure 3:**
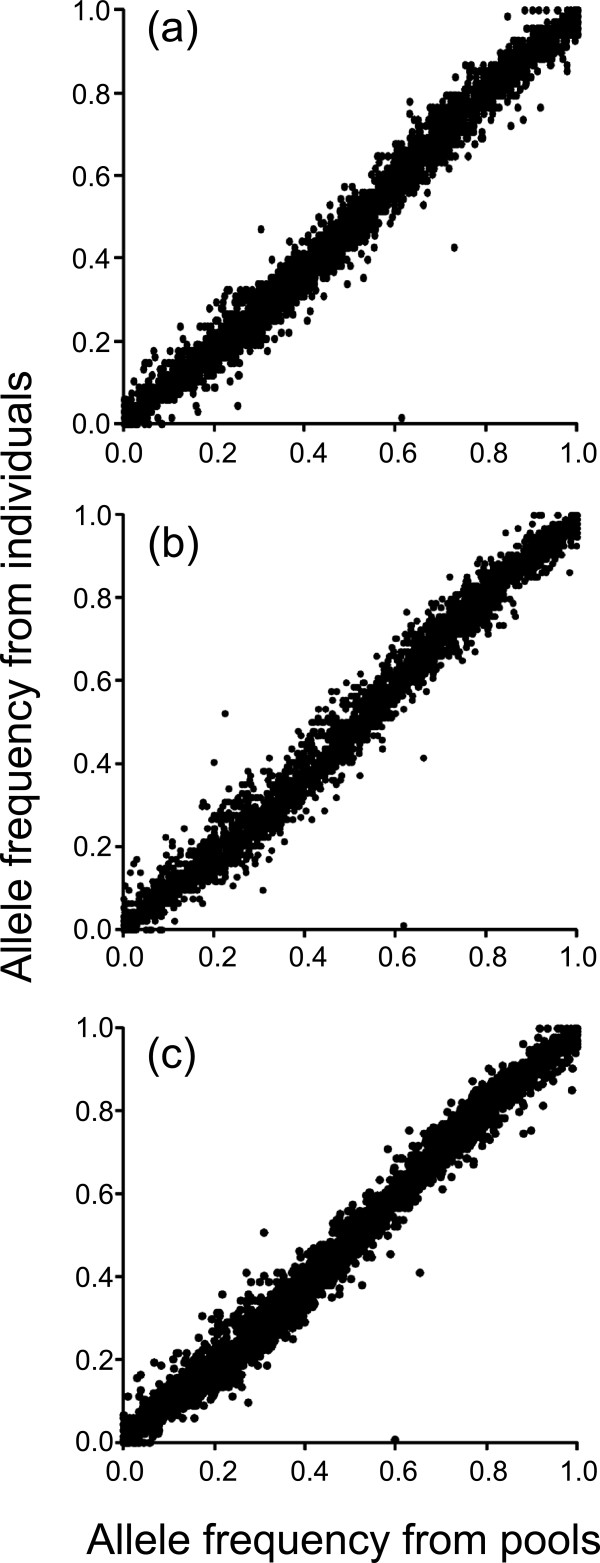
**Scatter plot of estimated allele frequencies from individual genotyping vs. pooled DNA.** ‘True’ allele frequencies from individual genotyping for Kola population were compared with estimated allele frequencies for three different pool sizes: (**a**) Kola-35 (n =35, *r* = 0.992), (**b**) Kola-50 (n = 50; *r* = 0.991) and (**c**) Kola-70 (n = 70; *r* = 0.992).

We observed very high concordance between allele frequency estimates derived from DNA pools and from individual genotyping (Pearson’s *r* = 0.991 – 0.992, all tests, *P* < 0.0001, Figure 3). This demonstrates the accuracy of the DNA pooling approach in Atlantic salmon and is consistent with earlier studies in other species using Illumina bead-array platform. For example, high correlation between allele frequency estimates derived from individual genotyping and DNA pools have been observed in humans (Pearson’s *r* = 0.969) and cattle (Pearson’s *r* = 0.992 – 0.994) [18,33]. The number of individuals in the DNA pool had only a minor effect on the allele frequency estimation (Figure 3) as the error between true and estimated allele frequencies was small and similar for all three pool sizes (median error = 0.023 – 0.025, Figure 2). Therefore, our results suggest that it is possible to obtain accurate allele frequency estimates using DNA pools consisting of relatively small number of individuals (n ≥ 35). However, larger pool sizes should be always preferred over small ones as small number of individuals may not be representative of the whole population.

### Quality control

One of the important parameters for accurate determination of genotypes and subsequent allelotyping is cluster separation score that quantifies the discrimination between genotype clusters for particular SNP (see Additional file 1: Figure S1b, c, d). Since the heterozygous cluster can be indistinguishable from one or both homozygous clusters for SNP with low cluster separation score, exclusion of loci demonstrating low cluster separation scores has been often applied [34,35]. To date, most of the studies have used a cluster separation score cut-off <0.35 to exclude low quality SNPs e.g. [36,37]. Based on visual inspection of SNP clusters in Atlantic salmon, however, cut-off value of 0.4 was chosen to efficiently exclude SNPs showing ambiguous genotype classes. This resulted in selection of 3631 out of 3897 markers for subsequent analysis. As expected, the error in allele frequency estimates of SNPs having cluster separation score < 0.4 was higher compared to SNPs with cluster separation score > 0.4 (Mann–Whitney *U* test, both for array and pool replicates, *P* < 0.0001) (see Additional file 1: Figure S2a, b). Moreover, the correlation between allele frequency estimates derived from three DNA pools and from individual genotyping for SNPs demonstrating low cluster separation scores (< 0.4) was lower than for markers with cluster separation scores > 0.4 (Pearson’s *r* = 0.960 – 0.969 vs. Pearson’s *r* = 0.991 – 0.992). In addition, the estimated variation of theta was negatively correlated with the cluster separation score both for array (Pearson’s *r* = − 0.346, *P* < 0.0001) and pool construction (Pearson’s *r* = − 0.246, *P* < 0.0001) replicates (see Additional file 1: Figure S3a, b).

While application of QC filter based on cluster separation excludes SNPs having low quality genotypes, it is not able to remove all loci showing relatively high variation in allele frequency estimates (see Additional 1: Figure S3a, b). Therefore, application of additional QC filters, e.g. based on comparisons between ‘true’ and estimated allele frequencies or based on combination of variation in allele frequency estimates and heterozygosity have been suggested e.g. [28,36,37].

**Figure 4:**
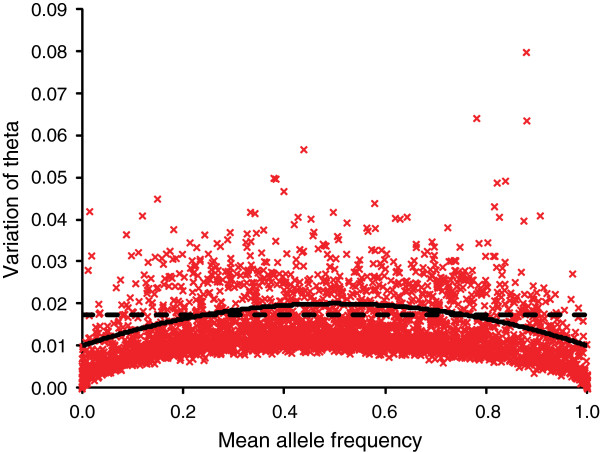
**A plot of mean estimated allele frequencies across 14 populations against array-variation.** Solid and dashed lines indicate the boundaries of spherical and uniform cut-offs, respectively.

Here, we tested two alternative QC filters (uniform and spherical cut-off) that use heterozygosity and variation in allele frequency estimates (Figure 4). This resulted selection of 2879 vs. 2880 loci for uniform and spherical cut-off, respectively (Table 2). Majority of loci (2777) that passed both filters were the same (Figure 4). However, spherical filtering is expected to be more useful than uniform cut-off as it retains larger proportion of polymorphic loci with mean allele frequency 0.2 – 0.8 across populations, while uniform filter increases the proportion of less variable loci (Table 2, Figure 4, Additional 1: Figure S4). Therefore, for identification of reliable and informative SNPs, application of spherical filter is preferable over uniform since it effectively excludes loci with relatively high error rate compared to the information content.

**Table 2:** Number of loci retained after applying spherical or uniform QC filtering of 3631 SNPs

**Filter**	**Mean allele frequency across 14 populations**					
	< 0.1	0.1 – 0.4	0.4 – 0.6	0.6 – 0.9	> 0.9	Total
*Before filtering*	326	1110	845	1044	306	3631
*Spherical*	275	877	690	787	251	2880
*Uniform*	308	860	639	785	287	2879

## Conclusions

This study tested the effectiveness of DNA pooling to obtain accurate allele frequency estimates for large number of Atlantic salmon populations using an Illumina SNP-chip. We demonstrated that pooled DNA approach provides a reliable, accurate and cost-effective means for obtaining genome-wide allele frequency estimates for multiple populations. We proposed a novel quality control filter based on spherical cut-off which enables efficient exclusion of loci showing high error rate and minor allele frequency close to zero. Our results indicate that even relatively small DNA pools (35 individuals) provide accurate allele frequency estimates for a given sample. Despite of higher levels of variation associated with array replicates compared to pool construction we suggest that both sources of variation should be taken into account. Taken together, this study demonstrates that DNA pooling allows fast and high-throughput determination of allele frequencies in Atlantic salmon enabling cost-efficient identification of informative markers for discrimination of salmon populations at various geographical scales, as well as identification of loci controlling ecologically and economically important traits. Moreover, the main findings of our study based on Atlantic salmon SNP-chip were in line with those observed for human SNP-chips, and thus the technical approaches described herein are encouraging for employing allelotyping approach in other species using Illumina SNP-chips or other SNP genotyping systems and arrays.

## Methods

### DNA samples

In total, 927 Atlantic salmon individuals representing 19 populations from Northern Europe were used for individual genotyping and/or construction of DNA pools (Table 1). Tissue samples (fin clips) were collected from juveniles during 2006 – 2010 and preserved in ethanol. Total genomic DNA was extracted according to Elphinstone et al. [38] or using Qiagen DNeasy 96 Blood & Tissue kits (Qiagen™) following manufacturer’s recommendations.

### Quality control of DNA extracts

Prior to pool construction, quality control of individual DNA extracts was performed in two steps. First, samples were examined for degradation by visual inspection on 1% agarose gels. Samples containing low molecular weight DNA (indicative of degradation) were excluded from further analysis. Each extract was then tested for contamination (the presence of DNA from multiple individuals) by screening individual samples using 18 microsatellite loci [39]; V. Wennevik, unpublished data] and only non-contaminated Atlantic salmon samples were selected for further analysis.

### Construction of DNA pools and SNP genotyping

In total, 56 DNA pools were constructed using individuals from 14 Atlantic salmon populations (Table 1). The adjustment of DNA concentration was carried out in two steps. The initial concentration of DNA samples was first adjusted to 20 ng/μl, measured in duplicate with the NanoDrop™ 1000 (Thermo Scientific) and subsequently diluted to 10 ng/ul. Individual DNA samples were pooled (50 ng per individual) and subsequently concentrated using a DNA concentrator Eppendorf 5301. The final concentration of the pools was adjusted to 50 ng/μl. Constructed DNA pools were analyzed using an Atlantic salmon Illumina SNP-chip [29,30] at the Centre for Integrative Genetics (CIGENE), Norway. In addition, 106 salmon samples used in pool construction were genotyped individually to guide cluster positioning and to obtain the ‘true’ allele frequency for each locus for the population from the River Kola (Table 1).

### Quality control

Genotyping of the 106 individual samples was performed using Genotyping module v. 1.9.4 (Genome Studio software v. 2011.1, Illumina Inc.), only those samples with > 97% call rates were included when calculating ‘true’ allele frequencies. SNPs with call rates < 95% (i.e. the proportion of individual samples successfully genotyped in a locus) were eliminated from the data set. Thresholds for quality control (QC) filtering were determined as in Murray et al. [37] and for estimation of allele frequencies from DNA pools, SNPs with cluster separation scores ≤ 0.4 were excluded.

### Estimation of allele frequencies in a pooled DNA samples

In Illumina genotyping, the genotype is assigned after converting raw color signal data into a theta value which ranges from 0 to 1 and reflects the relative signal contribution for the 2 alternate alleles. In theory, an individual homozygous for the B allele would have a theta value close to 1, an individual homozygous for the A allele a value close to 0 and a value of 0.5 would indicate a heterozygous genotype. However, in reality a SNP’s theta for genotype clusters (AA, AB and BB) may vary from 0, 0.5 and 1, therefore for estimation of allele frequency in a pooled sample, the theta value for each SNP is compared to the mean theta values for AA, AB and BB genotypes calculated by genotyping individual samples, i.e. the allele frequency of the DNA pool can be derived by applying correction algorithms from comparing pool-specific value of theta with the reference values of theta from individual genotyping data e.g. [40,41].

To obtain the allele frequency estimate for allele B in the pool *B*_*pool*_ Sample position of each pool along the axis of normalized theta values were compared to the reference values of AA, AB and BB genotype cluster positions for each SNP (reference values of theta) as in Janicki & Liu [41].

The following equations were applied [41]:

$$\begin{array}{l} \text{if}\;\theta_{\text{pool}}\leq \theta_{\text{AA}},\text{then}\;B_{\text{pool}}=0\;\text{or}\\ \text{if}\;\theta_{\text{AA}}<\theta_{\text{pool}}<\theta_{\text{AB}}\text{then}\;B_{\text{pool}}\\ \;=0.5\times \left(\theta_{\text{pool}}-\theta_{\text{AA}}\right)/\left(\theta_{\text{AB}}-\theta_{\text{AA}}\right)\;\text{or}\\ \text{if}\;\theta_{\text{AB}}<\theta_{\text{pool}}<\theta_{\text{BB}},\text{then}\;B_{\text{pool}}\;\\ \;=0.5+0.5\times \left(\theta_{\text{pool}}-\theta_{\text{AB}}\right)/\left(\theta_{\text{AB}}-\theta_{\text{AB}}\right)\;\text{or}\\ \text{if}\;\theta_{\text{pool}}\geq \theta_{\text{BB}},\text{then}\;B_{\text{pool}}=1,\;\text{where} \end{array}$$

*θ*_*pool *_is the sample position and *θ*_*AA*_*, θ*_*AB*_*, θ*_*BB*_ are means of the cluster positions of the corresponding reference genotypes along the axis of normalized theta values. The frequency of allele A was calculated as *A*_*pool *_= 1–*B*_*pool*_.

Reference values for AA, AB and BB genotype positions along the axis of normalized theta values were obtained from individual genotyping of 300 Atlantic salmon specimens genotyped in previous studies by CIGENE. As this data did not include samples from all the populations used to construct the DNA pools, the mean cluster position values were also derived from the genotype classes of 106 individuals originating from 8 populations across the study area (Rivers: Alta, Laukhelle, Iesjoki, Kola, Varzuga, Onega, Pechora Unya and Narva). For subsequent analyses, however, reference values of theta provided by CIGENE were used.

The accuracy of allele frequency estimates was quantified as an absolute difference between allele frequencies derived from individual genotypes (referred to as ‘true’) and allele frequencies estimated from DNA pools from the River Kola population (35, 50 and 70 individuals per pool).

### Estimation of array- and pool-construction variation

To estimate the within-pool variation of theta, replicates of the same DNA pool were run on different arrays (array replicates, as in Earp et al. [23]) (Table 1). To assess the variation in theta values introduced by pool construction, independently constructed pools consisting the same DNA extracts were run on same array (pool construction replicates, as in Earp et al. [23]) (Table 1). To evaluate the effect of number of individuals in the DNA pool on allele frequency estimation, DNA pools with varying number of individual DNA extracts were constructed (Table 1).

Variation of theta within a SNP locus was estimated similar to Macgregor [31]. The array-variation was calculated as the mean difference of all possible pair-wise comparisons of theta values among technical replicates of the same pool allelotyped on different arrays. The pool-construction variation was calculated as the mean difference of all possible pair-wise comparisons of theta values among technical replicates of the independently constructed DNA pools containing same individuals allelotyped on the same array.

## Competing interests

The authors declare that they have no competing interests.

## Authors’ contributions

M.O. constructed the DNA pools, performed the data analysis and lead in drafting the manuscript. J.-P.V. and A.V. designed the study and significantly contributed to the data analysis and the writing of the manuscript. V.W. took part in designing the study, provided microsatellite data and together with E.N. and S.P. collected biological samples. M.K. performed SNP array screening and initial data analysis. All authors read and approved the final manuscript.

## Supplementary Material

Additional file 1: Figure S1 Example of SNP loci failed to pass QC: a) call rate < 95%; b) cluster separation < 0.40; and SNP loci met QC requirements: c) cluster separation = 0.41 and d) call rate 100%, cluster separation = 1.00. Figure S2. Box-plot showing estimated variation of theta in two sets of SNPs with cluster separation score < 0.4 and > 0.4 for (a) array and (b) pool construction replicates (both tests, Mann–Whitney *U* test, *P* < 0.0001). Horizontal line, grey square, whiskers, open circles, and stars indicate median, 25th and 75th quartiles, non-outlier range, outliers and extreme outliers, respectively. Figure S3. A significant negative correlation between (a) array-(Pearson’s *r* = − 0.346, *P* < 0.0001) and (b) pool-construction (Pearson’s *r* = − 0.246, *P* < 0.0001) variation and cluster separation scores. Figure S4. Proportion of loci remained in each allele frequency class after application of (a) uniform and (b) spherical filter. Click here for file
