# Supplementary material for: Cost-effective genome-wide estimation of allele frequencies from pooled DNA in Atlantic salmon (Salmo salar L.)
Source: BMC Genomics. 2013 Jan 16;14:12. doi: 10.1186/1471-2164-14-12 (PMC3575319; doi:10.1186/1471-2164-14-12)
Supplement: Additional file 1: Figure S1 — Example of SNP loci failed to pass QC: a) call rate < 95%; b) cluster separation < 0.40; and SNP loci met QC requirements: c) cluster separation = 0.41 and d) call rate 100%, cluster separation = 1.00. Figure S2. Box-plot showing estimated variation of theta in two sets of SNPs with cluster separation score < 0.4 and > 0.4 for (a) array and (b) pool construction replicates (both tests, Mann–Whitney U test, P < 0.0001). Horizontal line, grey square, whiskers, open circles, and stars indicate median, 25th and 75th quartiles, non-outlier range, outliers and extreme outliers, respectively. Figure S3. A significant negative correlation between (a) array-(Pearson’s r = − 0.346, P < 0.0001) and (b) pool-construction (Pearson’s r = − 0.246, P < 0.0001) variation and cluster separation scores. Figure S4. Proportion of loci remained in each allele frequency class after application of (a) uniform and (b) spherical filter. [file 1471-2164-14-12-S1.docx]

Figure S1. Example of SNP loci failed to pass QC: a) call rate < 95%; b) cluster separation < 0.40; and SNP loci met QC requirements: c) cluster separation = 0.41 and d) call rate 100%, cluster separation = 1.00.





Figure S2. Box-plot showing estimated variation of theta in two sets of SNPs with cluster separation score < 0.4 and > 0.4 for (a) array and (b) pool construction replicates (both tests, Mann-Whitney U test, *P* < 0.0001). Horisontal line, grey square, whiskers, open circles, and stars indicate median, 25th and 75th quartiles, non-outlier range, outliers and extreme outliers, respectively.





Figure S3. A significant negative correlation between (a) array- (Pearson’s *r* = - 0.346, *P* < 0.0001) and (b) pool-construction (Pearson’s *r* = - 0.246, *P* < 0.0001) variation and cluster separation scores.





Figure S4. Proportion of loci remained in each allele frequency class after application of (a) uniform and (b) spherical filter.
